# The Diagnostic Challenge of Cystic Echinococcosis in Humans: First Assessment of Underreporting Rates in Mongolia

**DOI:** 10.3390/tropicalmed9070163

**Published:** 2024-07-19

**Authors:** Bolor Bold, Christian Schindler, Uranshagai Narankhuu, Agiimaa Shagj, Erdenebileg Bavuujav, Sonin Sodov, Tsogbadrakh Nyamdorj, Jakob Zinsstag

**Affiliations:** 1 National Center for Zoonotic Disease, Ulaanbaatar 18131, Mongolia; uranshagai@nczd.gov.mn (U.N.); agii.agiimaa@yahoo.com (A.S.); tsogbadrakh@nczd.gov.mn (T.N.); 2 Swiss Tropical and Public Health Institute, 4123 Allschwil, Switzerland; christian.schindler@swisstph.ch (C.S.); jakob.zinsstag@swisstph.ch (J.Z.); 3 Department of Public Health, Faculty of Medicine, University of Basel, 4001 Basel, Switzerland; 4 School of Global Health, Chinese Centre for Tropical Diseases Research, Shanghai Jiao Tong University School of Medicine, Shanghai 200025, China; 5 National Institute of Parasitic Diseases, Chinese Center for Disease Control and Prevention, Shanghai 200025, China; 6 Mongolian Society of Diagnostic Ultrasound, Ulaanbaatar 210648, Mongolia; erdenebileg32.eb@gmail.com (E.B.); sonin.sodov@gmail.com (S.S.)

**Keywords:** cystic echinococcosis, Mongolia, clinical guidelines, burden of disease, *Echinococcus granulosus*

## Abstract

Cystic echinococcosis (CE), caused by the larval stage of *Echinococcus granulosus*, is significantly underreported in Mongolia due to geographical remoteness, a lack of early diagnostics, and poor clinical management. This study aimed to provide a more accurate estimate of CE in Mongolia by comparing data from surgical (reported) and diagnosed (unreported) cases and assessing the challenges faced by rural doctors in disease management and surveillance. We collected data on surgical cases hospitalized between 2006 and 2016 and newly diagnosed CE cases in 2016 from eight provinces. Using a quasi-Poisson regression model, we extrapolated the collected data to estimate the number of diagnosed cases for the entire country. Additionally, forty health professionals from all 21 provinces rated local clinical management for CE through a questionnaire. The results reveal that surgical cases (2.2 per year) represent only one-eighth of diagnosed cases (15.9 per year). The laboratory facilities, disease reporting, and cyst classification usage scored below 2. These results highlight the significant underreporting of CE in Mongolia and urge human and animal health experts, along with policymakers, to invest in combating CE, particularly in remote provincial areas. This study also emphasizes the need for standard clinical management involving cyst classification according to the WHO-IWGE and seamless integration of CE reporting and monitoring mechanisms, which can significantly contribute to the national and global burden estimation of CE.

## 1. Introduction

Cystic echinococcosis (CE), caused by the larval stage of *Echinococcosis granulosus*, is a zoonotic disease with a substantial global impact on both human and animal health sectors [1,2]. Humans and intermediate host animals can get the infection by ingesting parasite eggs found in the feces of dogs or other canine species, which harbor the adult stage of the parasite in their intestines [3]. The ingested eggs can develop into cysts filled with parasite larvae, primarily affecting the liver and lungs, leading to the main clinical symptoms. The progression of symptoms in humans is chronic and can take months to years until diagnosis, depending on the cyst location, size, numbers, and the host’s immune reactivity [4,5].

Ultrasound serves as the gold standard for diagnosing, staging, and monitoring CE cysts, especially in abdominal sites [6,7]. Cyst staging of CE based on ultrasound is pivotal for clinical decision-making for uncomplicated liver CE cysts [5,8]. Gharbi et al. introduced the initial widely accepted cyst classification of CE in 1981 [9]. Although several classifications emerged thereafter, their adoption was limited. In 1994, due to the introduction of new potential treatments for CE, including albendazole and percutaneous treatment, the World Health Organization-Informal Working Group on Echinococcosis (WHO-IWGE) started to develop an internationally standardized ultrasound classification to establish universal application, replace the diverse range of classifications previously used, and guide clinical decision-making in treating CE patients [7,10,11,12]. The four treatment modalities that are specified in the WHO-IWGE are medical (albdendazole alone), percutaneous treatment such as Puncture, Aspiration, Injection, Re-aspiration (PAIR), surgical treatment for active cyst stages CE1 to CE3b, and ‘watch and wait’ for inactive cyst stages CE4 and CE5 [13,14,15,16]. However, an ultrasound scan can miss small cysts and lung cysts, and access to high-quality devices and expertise in recognizing CE characteristics is limited, especially in remote low- and middle-income countries [12,17].

Serology serves as a complementary method in addition to imaging. While the sensitivity and specificity of various antigens have been established, current assays still face challenges in standardization and accuracy [18]. Debates persist regarding their clinical diagnostic and screening utility, with serodiagnostic performance influenced by factors like cyst location, stage, and size, while these variables remain inadequately assessed to date [19,20]. A recent review confirmed that cyst staging significantly influences the sensitivity of ELISA tests, with sensitivity ranging from 60 to 90% for CE1–CE3 stages but dropping below 50% for CE4, CE5, and inactive cysts [8].

The disease mostly affects rural and farming communities due to their closeness to host animal populations [21,22]. In remote settings, expertise in ultrasound diagnosis and management is limited, and conventional serology techniques are lacking due to inadequate laboratory facilities and personnel in remote areas [17,23]. These conditions lead to underdiagnosis, underreporting, and a large number of individuals with CE remaining undetected until the disease progresses to an advanced stage, resulting in a significant underestimation of the disease’s global prevalence [24,25,26].

The current global disease burden of CE is estimated to be 184,000 disability-adjusted life years (DALYs), and this number can rise to more than 1 million if underreporting rates are adjusted based on a few available population surveys [1,2,25,26]. The complex challenge of quantifying the prevalence of CE has been prominently discussed in the recent WHO Neglected Tropical Disease roadmap 2021–2030 [27]. To overcome this challenge, there is a strong emphasis on establishing efficient diagnostic tools that are easily applicable in remote settings and on successfully implementing standardized guidelines for clinical management. This will greatly impact our understanding of the true disease distribution [17,24].

CE is endemic in Mongolia. The country has a large and widely distributed host animal population, as well as strong behavioral risks, while a high degree of unregulated slaughtering has been ongoing with no control actions over the last three decades [28,29,30]. A crucial detrimental factor for the control of CE was the sudden privatization of the veterinary sector following the economic collapse of the Soviet Union [29,31]. Currently, rural populations have very limited access to health services for CE. Each of the 21 provinces of Mongolia has one secondary-level hospital, the Provincial General Hospital (PGH), where radiologists initially detect or suspect CE cases using ultrasonography imaging [32]. These patients, except for fully calcified cases, are then referred to tertiary hospitals, located in the capital city of Ulaanbaatar, since there are no treatment options available at PGHs. Examinations of patients at PGHs often go unreported in electronic registries, and paper-based records of ultrasonography lack consistent information. Therefore, the current prevalence estimate of CE is based primarily on the number of cases that received surgical treatment, almost exclusively provided by the three national hospitals located in Ulaanbaatar city [32,33]. The challenge of finding reliable data on cases diagnosed at PGHs is the main cause of the significant underreporting [28,30,32]. Our study aims to provide a more accurate estimate of CE by establishing the underreporting rate from a comparison of data from surgical (reported) and diagnosed (unreported) cases, and to understand the challenges in clinical management that contribute to this lack of reporting through health professionals’ questionnaires.

## 2. Materials and Methods

### 2.1. Ethical Statement

This study was approved by the Medical Ethics Committee of Mongolia, the World Health Organization (WHO) Research Ethics Review Committee (ERC), and the Ethics Committee of North-Western and Central Switzerland (EKNZ 2014-240). Permission to access the hospital data and statistical data was obtained. Verbal and written informed consent were given by each health professional. The collected data were only available to the study team. All patient data were rendered anonymous prior to further analysis. 

### 2.2. Data Collection

To estimate the underreporting rate, we divided the CE cases into two different categories, depending on the level of health service received and the situation of their registration:

#### 2.2.1. Surgical Cases

We collected data of patients hospitalized between 2006 and 2016 in the surgical departments of national hospitals who were identified with a discharged with diagnosis under the ICD 10 code for CE, ranging from B67.1 to B67.9. These data were retrieved from the digital database archive of the Center for Health Development, Ministry of Health, Mongolia. Information extracted included patient age, sex, registration number, residential province, admission date, hospital name, and treatment category. 

#### 2.2.2. Diagnosed Cases

Cases diagnosed by ultrasonography at PGHs but which have not yet received any treatment at a tertiary hospital are termed diagnosed cases. Except for fully calcified cases, diagnosed cases are typically referred to tertiary hospitals since there are no treatment options available at PGHs. Obtaining data records for these cases is highly challenging due to inconsistencies in reporting and the lack of maintenance of paper-based records. Therefore, we collaborated with radiologists from PGHs in eight provinces (around 40% of all PGHs) to enter the newly diagnosed cases from one year (2016) into our online data collection tool developed only for this study. The eight surveyed PGHs are located in the following eight provinces: Umnugobi and Gobi-Altai provinces in the arid south, suitable for camel and goat farming and registering the highest surgical cases in the last decade; Uvurkhangai, Arkhangai, Zavkhan, and Khentii in the central flat steppes, ideal for sheep grazing; and Khubsgul in the mountainous, forested north. These provinces have populations ranging from 50 to 100,000 and are predominantly inhabited by nomadic farming communities with high livestock populations and numerous free-roaming dogs, which facilitate the spread of CE. 

To reduce misdiagnoses, all participating radiologists had a minimum of 2 years of radiology training, and only those with over 10 years of experience were recruited. Anonymized patient information, including age, sex, cyst location, imaging features, and treatment recommendations, was recorded in the online system. To avoid duplicating cases, doctors entered special registration numbers for patients, which helped to prevent repeated cases. Moreover, radiologists created a paper version of the record for later double entry by the local epidemiologists. We excluded X-ray diagnoses due to their low sensitivity in detecting CE, so pulmonary cases were not included in our survey. Since fully calcified cases do not need treatment and are not referred, we excluded all cases diagnosed as having full calcification.

### 2.3. One Round Delphi Survey of Healthcare Professionals

The survey was conducted adjunct to the national stakeholder seminar on CE, held on 18 September 2016. Participants were invited to complete the survey following their attendance at the seminar, with the aim of gathering expert opinions on clinical management and diagnostic challenges of CE in provincial hospitals. We invited one radiologist from each PGH and one epidemiologist from the local zoonotic center in each province (infection specialists at the PGH were invited for the provinces that had no local zoonotic center) to answer the questionnaire. A total of 17 radiologists from 17 provinces, 21 epidemiologists from 21 provinces, and 2 epidemiologists from Ulaanbaatar city participated in this survey.

A questionnaire comprising 20 questions assessed the clinical management of CE at the PGH, focusing on diagnosis, treatment, and reporting. Participants rated each indicator on a scale from 0 to 6, with 0 indicating the worst score and 6 indicating the best score. We categorized the questions into three sections according to their related topics: diagnosis, treatment, and surveillance. Here, we provide the main contents of these three sections (the full version is in Supplementary Document S1): (i) Diagnosis: 1.1 Availability of Ultrasonography, 1.2 Adequacy of Imaging Doctors (2 years of training), 1.3 Adequacy of Imaging Doctors (3 months of training), 1.4 Use of Clinical Guidelines, 1.5 Doctor’s Ability to Identify CE, 1.6 Availability of Serological Kits, 1.7 Availability of Parasitological Labs 1.8 Availability of Parasitologists, 1.9 Availability of the Histological Lab, 1.10 Availability of Histologists; (ii) Treatment: 2.1 Use of Cyst Classification, 2.2 Availability of Albendazole, 2.3 Knowledge of Albendazole Treatment, 2.4 Frequency of Monitoring Check-ups; 2.5 Frequency of Follow-up Visits; (iii) Surveillance: 3.1 Use of Digital Registration; 3.2 Consistency of Referral Registration; 3.3 Consistency of Treatment Registration; 3.4 Consistency of Notifiable Disease Reporting (NDR); and 3.5 Consistency of Reporting to Zoonotic Center.

### 2.4. Data Analysis

#### 2.4.1. Estimating the Underreporting Rate of CE

We first removed all calcified cases from our registry of diagnosed cases, as these do not require treatment, only monitoring. Additionally, pulmonary cases mostly cannot be identified through ultrasonography, so we disregarded them from the registry of surgical cases. The number of diagnosed but not yet surgically treated (non-surgical) cases in 2016 was calculated as the difference between the number of diagnosed cases and the number of surgical cases. These differences were fitted using a quasi-Poisson regression model, which included the natural logarithm of the average annual count of surgical cases from 2012 to 2016 in the respective province as an independent variable. The model was used to predict the number of diagnosed non-surgical cases in 2016 across other provinces.

For the eight provinces with recorded numbers $y_{i}$ of diagnosed non-surgical cases, the following empirical Bayes estimate was used instead of the observed number $y_{i}$:

(1) $$\hat{y_{i}^{EB}}=w_{i}\hat{y_{i}}+\left(1-w_{i}\right)y_{i}$$

where $y_{i}$ and $\hat{y_{i}}$ denote the recorded and fitted numbers of non-surgical cases in province i, respectively, and

(2) $$w_{i}=\frac{y_{i}}{y_{i}+{\left[SE\left(\hat{y_{i}}\right)\right]}^{2}}$$

In the other provinces, the number was predicted by $\hat{y_{i}}$. Similarly, an empirical Bayes estimate was computed for the average annual number of surgical cases in 2016 using the prediction provided by a quasi-Poisson regression model with a linear time trend variable fitted to the annual numbers of surgical cases between 2006 and 2016.

The numbers of surgical and non-surgical cases in 2018 were then estimated by multiplying their estimates for 2016 by the factor

(3) $$exp\left(2\hat{\beta }\right)$$

with $\hat{\beta }$ denoting the coefficient of the variable year in the aforementioned model.

Furthermore, 95% confidence intervals were computed for the estimated numbers of cases in 2018 by simulating the uncertainty around the point estimates. For this purpose, we distinguished between the number $n_{1}$ of surgical cases, the number $n_{2}$ of non-surgical cases in provinces without data on non-surgical cases, and the number $n_{3}$ of non-surgical cases in the eight provinces that provided data on these cases in 2016. The simulation involved adding a random term

(4) $$SE\left(\hat{y}\right)\cdot z_{1}$$

to the respective point estimate $\hat{y}$, with $z_{1}$ being sampled from the standard normal distribution, and then multiplying this sum by

(5) $$exp\left(2\hat{\beta }\right)\cdot \left(1+2SE\left(\hat{\beta }\right)\cdot z_{2}\right)$$

with $z_{2}$ being another standard normal random number. The standard error of the estimate of $n_{2}$ included two components: the uncertainty of the regression model and the uncertainty associated with the overdispersion of provincial counts.

In a further step, the estimated counts of surgical and non-surgical cases were divided by the proportion $p_{NP}$ of non-pulmonary surgical cases to obtain estimates of all clinical cases. To account for the statistical uncertainty introduced by this factor, these estimates were multiplied by the factor

(6) $$\;\;1-\frac{SE\left(p_{NP}\right)}{p_{NP}}\cdot z_{3}$$

with $SE\left(p_{NP}\right)$ denoting the standard error of $p_{NP}$ and $z_{3}$ being another standard normal random number.

After iterating these simulation steps 1,000,000 times, the 95% confidence limits were estimated by the 2.5th and 97.5th percentiles of the simulated values.

#### 2.4.2. Summarizing the Healthcare Professionals Survey

The survey results, rated on a Likert scale from 0 to 6, were aggregated and averaged to rank results from high to low scores to identify the most significant challenges in the clinical management of CE at the provincial (secondary) level.

## 3. Results

### 3.1. Estimated Cases of CE

A total of 446 surgical CE cases were reported between 2006 and 2016 in Mongolia. The mean age of cases was 28.3 (95% CI 26.4–30.3). The percentages of males and females were 44% and 56%, respectively. For the diagnosed cases, a total of 185 cases were detected with CE in the abdominal organs from eight provinces during 2016. The mean age of diagnosed cases was 57.9 (95% CI 55.3–60.6). The percentages of males and females were 30% and 70%, respectively. The estimated number of diagnosed (surgical and non-surgical) cases for 2018 is 476 (95% CI 387–570) (Table 1). The prevalence (per 100,000 people) based on the estimated number is 2.2 (95% CI 1.8–2.7) for surgical cases and 15.9 (95% CI 12.9–19.0) for diagnosed cases (Table 1).

### 3.2. Survey Results from Healthcare Professionals

**Figure 1:**
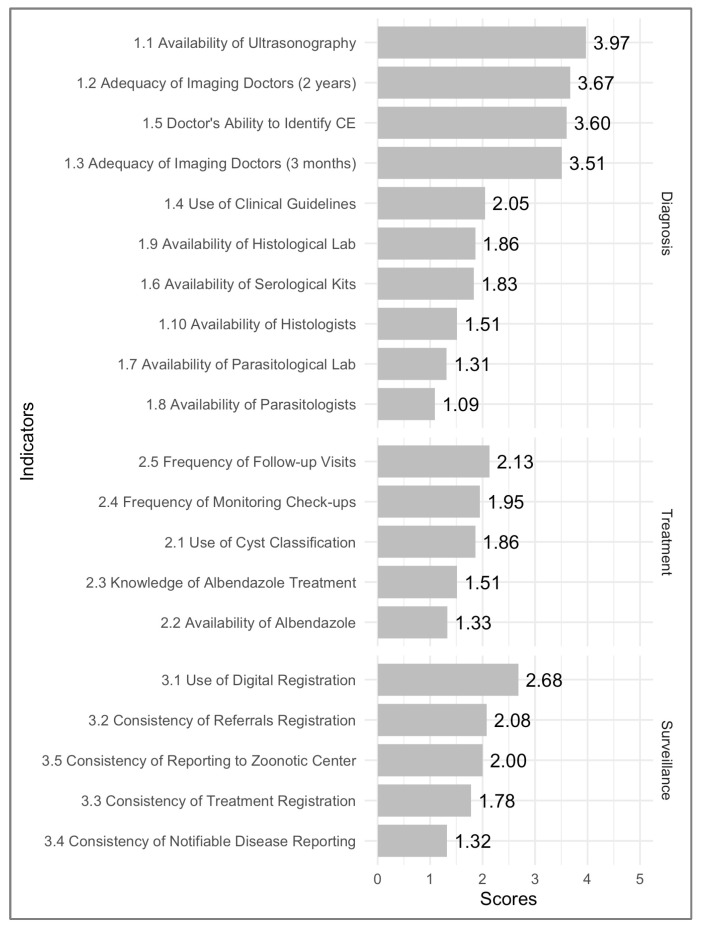
Average adequacy scores for the clinical management of CE at PGH by healthcare professionals across the 21 provinces of Mongolia.

The results from the health professionals survey are shown in Figure 1, which shows that in the diagnosis category, the highest score was for the Availability of Ultrasonography, which received a rating of 3.97. The lowest score in this category was for the Availability of Parasitologists, with a rating of 1.09. All the scores related to laboratory capacities are low, ranging between 1.31 and 1.83. The next lowest score is for the Use of Clinical Guidelines, which has a rating of 2.05. In the treatment category, the highest score was for the Frequency of Follow-up Visits, rated at 2.13, while the lowest score was for the Availability of Albendazole, with a rating of 1.33. Except for the Frequency of Follow-up Visits, all scores in the treatment category are below 2. For the Surveillance category, the highest score was for the Use of Digital Registration, which had a rating of 2.68, and the lowest score was for the Consistency of Notifiable Disease Reporting (NDR), with a rating of 1.32.

Overall, the top three scores were for the Availability of Ultrasonography (3.97), the Adequacy of Imaging Doctors (2 years) (3.67), and the Doctors’ Ability to Identify CE (3.60). The lowest three scores were for Availability of Parasitologists (1.09), Availability of Parasitological Labs (1.31), and Consistency of Notifiable Disease Reporting (1.32).

## 4. Discussion

In this study, we aimed to address the underreporting of CE in Mongolia by comparing surgical and diagnosed cases, and to understand the challenges in the clinical management of CE patients in rural provinces through a survey of health professionals. Our results suggest that the current prevalence of CE based on surgical cases, which is 2.2 per year, might represent only one-eighth of the total number of diagnosed cases, estimated to be 15.9 per year. Additionally, healthcare professionals rated laboratory facilities, disease reporting, and the usage of cyst classification in clinical practice as poorly performing, with all aspects scoring below 2 out of 6. These results emphasize the need for the standardization of clinical management systems with cyst classification such as WHO-IWGE [10] and the seamless integration of CE reporting and monitoring mechanisms, which can significantly improve the underreporting situation of CE.

Estimating the global burden of CE is highly challenging, particularly as the disease primarily affects rural pastoral communities where residents live far from healthcare facilities [24,25,26]. In such settings, advanced diagnostic capacity is often limited. This is reflected in our survey of health professionals, where parasitological, histological, and serological laboratory facilities all scored below 2. The remoteness and diagnostic challenges are inherent aspects of the disease that are difficult to change [17,34]. In contrast, clinical management and surveillance systems can be improved with better communication and governance [7,12,27]. In our survey, CE reporting to the NDR system in Mongolia received the second lowest average score, despite the fact that such reporting should be mandatory for most infectious and zoonotic diseases. This is because CE cases are predominantly encountered by clinicians, particularly surgeons and radiologists, who often do not have direct contact with infection specialists or epidemiologists. In 2017, the Ministry of Health mandated CE reporting in the NDR, but this initiative eventually failed to yield results due to the absence of clear guidance on communication between clinicians and epidemiologists [32]. This experience underlines the importance of effective communication and dialogue between and within sectors for handling complex diseases such as CE [7].

Our survey indicates that Mongolian health specialists do not view a lack of ultrasound machines or technical ability to identify CE by ultrasound as the main issues; these aspects scored highest in adequacy with scores ranging from 3.60 to 3.97. Instead, they identify poor usage of cyst classification and inadequate disease monitoring as significant obstacles to effective clinical decision-making, both scoring lowest with 1.86–1.95. Implementing the current clinical guidelines from WHO-IWGE, which include cyst classification and stage-specific treatment algorithms, can significantly improve the situation [12,28,32]. In 2016, the National Center for Zoonotic Disease and the Mongolian Society of Diagnostic Ultrasound introduced the WHO-IWGE guidelines to Mongolian doctors. This initiative was implemented through multisectoral dialogue [32]. However, its effectiveness raises questions, given the country’s lack of the four treatment modalities outlined in the guidelines. Percutaneous treatments such as PAIR are not performed due to a lack of interventional radiologists in the country. Furthermore, albendazole supply is insufficient and costly [32]. This leaves surgery as the only remaining treatment option. In consequence, the utility of the WHO-IWGE guideline and its cyst classification remains limited. In fact, many endemic countries face similar challenges to Mongolia due to the absence of a standardized cyst classification system [24,35]. A recent review revealed that only 50% of the 173 studies selected for review used cyst staging in their reports, highlighting the global challenges in implementing cyst staging in clinical settings [8,36]. Significant progress has been made with the recent introduction of free albendazole distribution organized by the WHO. Such progress is crucial for the effective global implementation of the WHO-IWGE guideline, as it ensures non-surgical treatment at remote, local healthcare facilities, reducing the need to refer patients to advanced facilities, minimizing unnecessary surgeries and their subsequent complications and alleviating the economic burden of patients [27,37].

Resources for serological tests at the provincial level, scoring 1.83, were another challenge addressed by health professionals. Although currently available conventional serological methods, such as the ELISA test, are not yet fully reliable for diagnostic purposes, significant progress in their sensitivity and specificity has been made in recent years [18,19,20]. However, in Mongolia, where maintaining lab facilities and resources in remote areas is difficult, easily applicable tests such as Rapid Diagnostic Tests (RDTs) might be more practical at the provincial level. Recent reviews have shown that RDTs for active cysts demonstrate a sensitivity of 74% and a specificity of 96%, which are comparable to the ELISA test results of 69% sensitivity and 96% specificity [28,38]. Furthermore, current evaluations of serological analyses indicate that cyst staging is the only statistically significant factor for the accuracy of serological results, emphasizing the importance of cyst classification [28,32].

Based on our estimates, the prevalence could be approximately 16 cases per 100,000 people, which is eight times higher than the number of reported surgical cases. This does not imply that this is the true prevalence, but rather a more accurate estimate if passive surveillance were included in the CE. To make both of our data sources as comparable as possible, we did not account for non-abdominal and calcified cases in our estimation. Non-abdominal cases are unlikely to be detected at the PGH, and calcified cases would not be reported as surgical cases. Thus, the actual number of diagnosed cases is likely higher. In terms of the number of cases, regional differences may exist, as evidenced by a recent ultrasonographic survey in four Mongolian provinces showing prevalence variations from 2% to 13% [39]. We did not compare regional differences, focusing instead on underreporting due to issues in clinical management and surveillance systems. The robustness of our data collection is supported by the high qualifications of participating radiologists, who had a minimum of 2 years of radiology training and over 10 years of professional experience, ensuring accurate identification of CE.

Our study highlights the significant underreporting of CE in Mongolia, urging human and animal health experts, along with policymakers, to invest in combating CE, particularly in remote provincial areas. It emphasizes the need for further investigation at the population level within the One Health framework to determine the true extent of the disease burden and to obtain more accurate infection rates for control measures [40]. While the remoteness of affected communities, chronic latent symptoms, and lack of early detection methods pose inherent challenges that are difficult to overcome, improvements in clinical guidelines, cyst classification, and clear coordinated mechanisms between physicians and public health specialists for information exchange can significantly enhance disease management and surveillance. Furthermore, upgrading CE medical education for healthcare specialists and promoting better clinical management for CE in post-graduate meetings and conferences will greatly contribute to the sustainability of guideline usage. Authorities can work toward facilitating secondary health centers with early identification methods and non-surgical treatments. Our study provides valuable insights that are helpful to understand the challenges in implementing the WHO-IWGE guidelines and in disease reporting in low- and middle-income countries.

## Figures and Tables

**Table 1:** Predicted number of cases of CE for 2018.

Categories	Cases		Prevalence *	
	Mean	95% CI	Mean	95% CI
Surgical cases	67	55–80	2.2	1.8–2.7
Non-surgical cases	409	321–500	13.6	10.7–16.6
Total diagnosed cases	476	387–570	15.9	12.9–19.0

* Number of cases per 100,000 people.

## Data Availability

Data supporting the reported results are available from the National Center for Zoonotic Disease of Mongolia (NCZD) for researchers who meet the criteria for access to confidential data. Interested researchers can request access by contacting the director of the National Center for Zoonotic Disease of Mongolia at amgalanbayar.bandikhuu@nczd.gov.mn or nczd@nczd.gov.mn. Due to privacy and ethical restrictions, the data are not publicly available.

## Supplementary Materials

The following supporting information can be downloaded at: https://www.mdpi.com/article/10.3390/tropicalmed9070163/s1. Document S1: The full version of the questions in the survey of healthcare professionals.
