# Supplementary material for: The Diagnostic Challenge of Cystic Echinococcosis in Humans: First Assessment of Underreporting Rates in Mongolia
Source: Trop Med Infect Dis. 2024 Jul 19;9(7):163. doi: 10.3390/tropicalmed9070163 (PMC11281321; doi:10.3390/tropicalmed9070163)
Supplement: Supplementary file 1 [file tropicalmed-09-00163-s001.zip › tropicalmed-3078661-supplementary.pdf]

Evaluation of Clinical Management of Echinococcosis at Provincial (Secondary) Level Health Care Facilities of Mongolia

Thank you for participating in this evaluation of the clinical management of echinococcosis at the provincial (secondary) level in Mongolia. Please provide a rating between 0 and 6 for each of the following questions, where:

- 0 represents the worst possible rating (e.g., not available at all, never used, etc.)

- 6 represents the best possible rating (e.g., always available, always used, etc.)

Your responses will help us understand the current status and identify areas for improvement.

1. Questions related to diagnosis:

1.1. How would you rate the availability of ultrasonographic machines at your hospital?

1.2. How adequate do you find the number of doctors who specialized as imaging doctors at university for 2 years?

1.3. How adequate do you find the number of doctors who completed a 3-month course in diagnostic imaging?

1.4. How frequently are clinical guidelines/manuals used to diagnose cystic echinococcosis at your hospital?

1.5. How would you rate the ability to identify CE of the radiologist at your hospital?

1.6. How would you rate the availability of diagnostic kits for serological tests of cystic echinococcosis at your hospital?

1.7. How would you rate the availability of a parasitological laboratory at your hospital?

1.8. How would you rate the availability of parasitologists?

1.9. How would you rate the availability of a histological laboratory at your hospital?

1.10. How would you rate the availability of histologists?

2. Questions related to treatment:

2.1. To what extent is cyst classification used during diagnosis to optimize treatment options?

2.2. How would you rate the availability of albendazole medication in your province?

2.3. How would you rate the availability of doctors who have knowledge of using albendazole treatment for cystic echinococcosis?

2.4. What is the frequency of monitoring check-ups for diagnosed patients?

2.5. What is the frequency of follow-up visits after surgery?

3. Questions related to reporting and surveillance:

3.1. How extensively is a digital registration system used for cystic echinococcosis cases?

3.2. How consistently are patients who are referred to the next level hospital registered/reported?

3.3. How consistently are patients who have undergone cystic echinococcosis treatment registered/reported?

3.4. How consistently are cases reported to the notifiable disease registration (infectious disease department/ward) at your hospital?

3.5. How consistently are cases reported to the local zoonotic disease center?
